# The effect of MElatonin on Depressive symptoms, Anxiety, CIrcadian and Sleep disturbances in patients after acute coronary syndrome (MEDACIS): study protocol for a randomized controlled trial

**DOI:** 10.1186/s13063-017-1806-x

**Published:** 2017-02-23

**Authors:** Michael Tvilling Madsen, Anders Isbrand, Ulla Overgaard Andersen, Lars Juel Andersen, Mustafa Taskiran, Erik Simonsen, Ismail Gögenur

**Affiliations:** 1 Department of Surgery, Zealand University Hospital, University of Copenhagen, Lykkebaekvej 1, 4600 Koege, Denmark; 2 Department of Cardiology, Zealand University Hospital, Lykkebaekvej 1, 4600 Koege, Denmark; 3 Department of Cardiology, Holbaek Hospital, Smedelundsgade 60, 4300 Holbaek, Denmark; 4 Department of Cardiology, Zealand University Hospital, Roskilde, Koegevej 7-13, 4000 Roskilde, Denmark; 5 Psychiatric Research Unit, Region Zealand, Faelledvej 6, Bygning 3, 4. Sal., 4200 Slagelse, Denmark; 6 Institute of Clinical Medicine, Faculty of Health and Medical Sciences, University of Copenhagen, Copenhagen, Denmark

**Keywords:** Depression, Melatonin, Acute coronary syndrome, Anxiety, Sleep disturbances, Circadian rhythm

## Abstract

**Background:**

Depression following acute coronary syndrome (ACS) constitutes a serious and debilitating problem. Approximately one in five patients will develop significant depression following ACS and less severe depressive symptoms are even more frequent. Furthermore, anxiety symptoms and sleep-wake disturbances are frequent. The objective of the MEDACIS trial is to investigate whether prophylactic treatment with melatonin has a preventive effect on depression, depressive and anxiety symptoms, sleep, and circadian disturbances following ACS.

**Methods/design:**

“The effect of MElatonin and Depressive symptoms, Anxiety, CIrcadian and Sleep disturbances in patients after acute coronary syndrome” trial (MEDACIS) is a multicenter, double-blinded, placebo-controlled, randomized clinical trial. A total of 240 patients with ACS and no depressive symptoms will be included in the trial for treatment with either 25 mg melatonin or placebo for a 12-week period. Development and severity of depressive symptoms will be evaluated using Major Depression Inventory every 2 weeks with the purpose of investigating the potential preventive effect of melatonin on depressive symptoms.

**Discussion:**

Previously, only selective serotonin reuptake inhibitors (SSRIs) have been investigated in a primary preventive setup in patients following ACS. However, SSRIs are associated with several side effects. An ideal intervention would constitute the highest degree of prevention of depressive symptoms with the lowest risk of side effects. In this regard, melatonin may have advantages due to its low toxicity as well as its proven anxiolytic and hypnotic effects.

**Trial registration:**

ClinicalTrials.gov, Identifier: NCT02451293. Registered on 12 May 2015. EudraCT nr. 2015-002116-32.

**Electronic supplementary material:**

The online version of this article (doi:10.1186/s13063-017-1806-x) contains supplementary material, which is available to authorized users.

## Background

By the year 2030 it has been anticipated that depressive disorder and ischemic heart disease will be the first and second leading causes of disability in developed nations, respectively [1]. Worldwide, an estimated 7 million people or more each year suffer from myocardial infarction (MI) [2]. In Denmark in 2011, the incidence rate of acute myocardial infarction was 553 cases/100,000 person-years, a prevalence of 4570 cases/100,000 persons, and a mortality of 123 cases/100,000 persons [3]. The prevalence of major depressive disorder following acute coronary syndrome (ACS) has been reported to be approximately 19.8% ((CI) 19.1% to 20.6%) assessed by structured clinical interviews [4]. However, the prevalence varies according to the tool used to evaluate depression [4–6], e.g., if the most frequently used self-report instrument, the Beck Depression Inventory (BDI), is used the prevalence is 31.1% ((CI) 29.2% to 33.0%) [4]. However, a prevalence as high as 50.5% has been reported with other questionnaires [5].

Depression following ACS has been associated with a 2.25 ((odds ratio), CI 1.73 to 2.93) increase in all-cause mortality, and a 2.71 ((odds ratio), CI 1.68 to 4.36) increase in cardiac mortality within 24 months of the primary event [7]. Depression was recently categorized by the American Heart Association (AHA) as an independent risk factor for adverse medical outcomes in patients after ACS [6]. Furthermore, the AHA has made recommendations for implementing screening for depression in the post-MI period [8].

Anxiety symptoms following ACS have not received as much attention as post-ACS depression [9]. A mix of both depressive and anxiety symptoms tend to coexist in patients following ACS [10] and they are found in up to 90% of depressed patients following MI [11]. The prevalence of anxiety symptoms varies from 24 to 37% among hospitalized MI patients [12, 13], and has been found to be associated with a 1.47-fold ((odds ratio), CI 1.02 to 2.13) increase in all-cause mortality [14]. However, a more recent meta-analysis could not confirm [15] this in post-ACS patients and the relationship was thought to be attenuated by comorbid depression. Anxiety symptoms have been shown to reduce heart rate variability [16], activate the hypothalamic-pituitary-adrenal axis [17], and enhance fibrin turnover [18]. Furthermore, anxiety is an independent predictor of cardiac events following MI [11, 19].

Sleep disturbances are prevalent in cardiac patients [20] and sleep disturbances are an integral part of both depression and anxiety symptoms [21]. In fact, in relation to the pathophysiology of depressive disorders, sleep and sleep-wake disturbances have been identified as two of the most important contributing factors [22]. The prevalence of sleep disturbances in ACS patients has not received sufficient attention [20], and the primary focus of the medical literature has been on sleep apnea, because of the known relationship between sleep-disordered breathing and cardiovascular morbidity [23]. This lack of relevant medical literature is disturbing, especially seen in the light of poor sleep having been shown to be associated with a 2.5-fold increased risk of recurrent cardiac events in women after ACS [24].

There exists a complex relationship between depression, sleep and circadian disturbances [25], and residual sleep disturbances may remain despite treatment of depression [26]. Furthermore, cognitive behavioural therapy (CBT) and pharmacotherapy have been shown to be equally effective in treating sleep disturbances after remission from depression [26]. Both psychological and pharmacological interventions could potentially be warranted in patients suffering from both medical illness and depression [27]. As oppose to antidepressants, psychotherapy has been shown to have a small but significant effect in the prevention of depression after stroke [28]. However, a limitation of psychological interventions is that the therapist and patient cannot be blinded to allocation which increases the risk of bias. An intervention where blinding is possible and which can target both the depression and the anxiety as well as the circadian and sleep disturbances in patients after ACS would be of clinical interest.

**Fig. 1:**
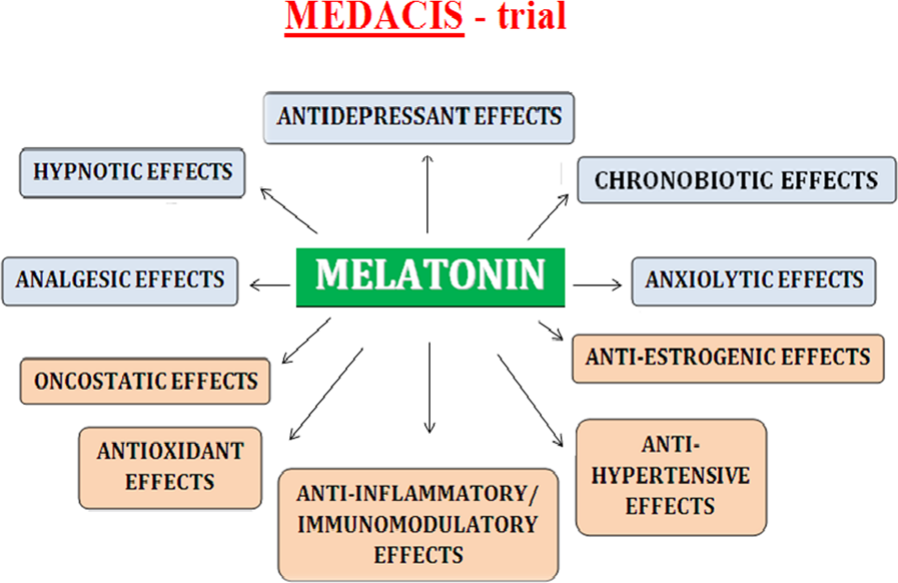
MElatonin on Depressive symptoms, Anxiety, CIrcadian and Sleep disturbances (MEDACIS) trial. Potential effects of exogenous melatonin intervention

One possible pharmacological intervention investigated in the current trial could be the endogenous hormone melatonin (ATC: N05CH01). Melatonin is secreted from the pineal gland in a circadian pattern, controlled by the endogenous circadian pacemaker in the suprachiasmatic nuclei [29]. The primary function of melatonin is to synchronize the circadian rhythm of the body [29]. However, melatonin is a multipotent hormone and affects multiple processes in the human body [30–36] (Fig. 1). It has been shown to prevent depressive symptoms over a 3-month period in patients with breast cancer [37]. In the same population, it was shown to improve sleep quantity and quality both during a 2-week and a longer-term period (3 months) [38, 39]. Meta-analyses have shown melatonin to be effective in the treatment of primary sleep disorder [40]; however, the effect in secondary sleep disorders is limited [41]. In a perioperative setting, melatonin treatment has been shown to have an anxiolytic effect [42]. Choosing the ideal dose of melatonin regarding its antidepressant effect is difficult as no ideal dose-response studies have been carried out on this indication. Typically, melatonin is administered in under 10-mg doses [43] which is the dose used for treatment of insomnia in patients aged over 55 years [44]. The melatonin agonist agomelatine is on the market for the treatment of depression, and choosing an equipotent dose of melatonin to this agonist has been suggested [30]. The pharmacodynamics and pharmacokinetics of agomelatine and melatonin are similar [30] and the induction dose of agomelatine is 25 mg. A dose of 25 mg was chosen in the current trial as a balance between the primary prophylaxis (treatment before incident disease) design and the nontoxic profile of melatonin [40, 41, 45].

### Aim

The primary aim of the current study is to investigate a possible prophylactic antidepressant effect of melatonin in patients following ACS. Secondary aims will be to investigate whether melatonin will have effect on anxiety symptoms, sleep disturbances and circadian rhythm.

## Methods/design

**Fig. 2:**
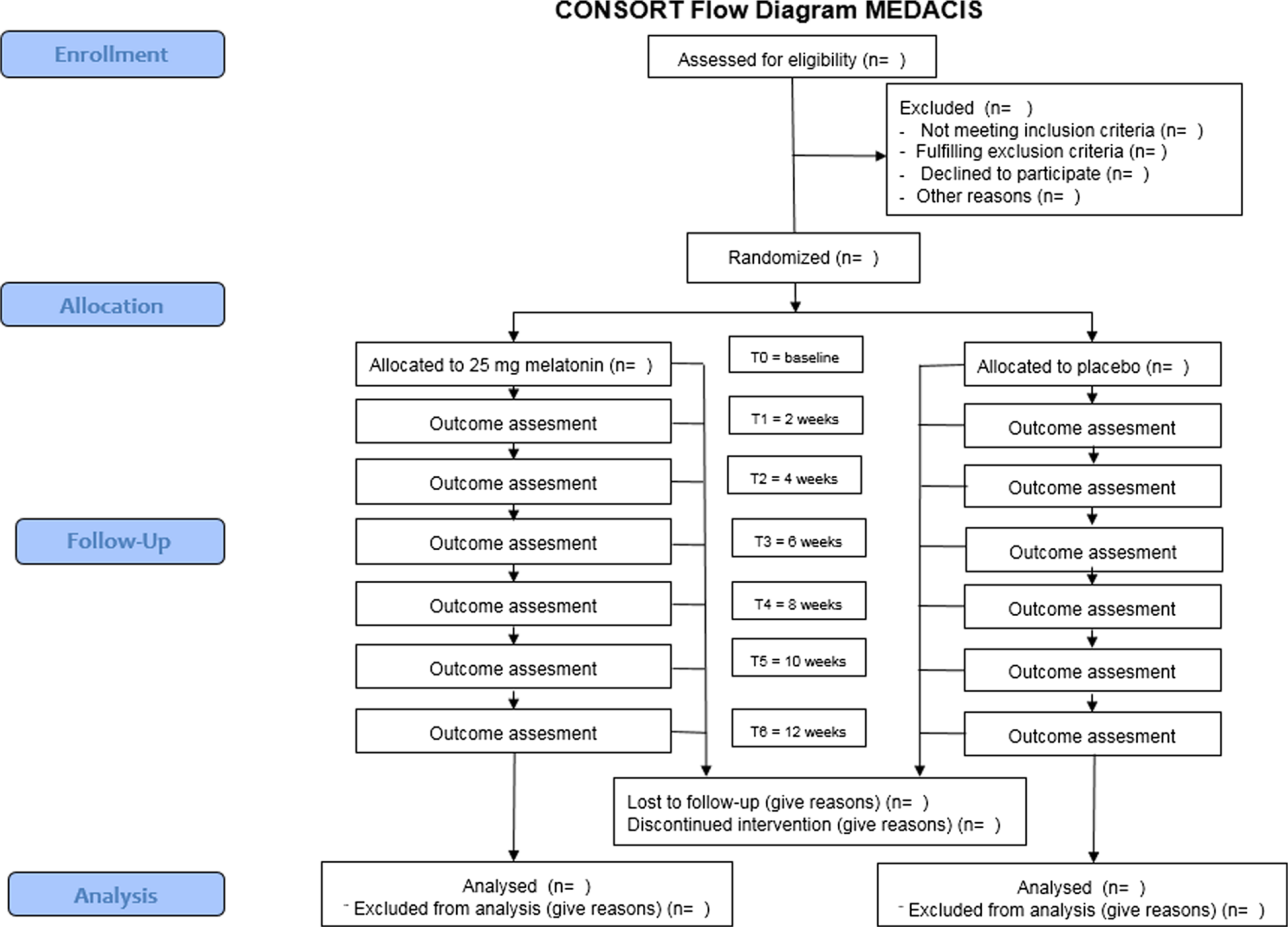
MElatonin on Depressive symptoms, Anxiety, CIrcadian and Sleep disturbances (MEDACIS) CONSORT diagram. Consolidated Standards of Reporting Trials (CONSORT) diagram for the MEDACIS trial

**Fig. 3:**
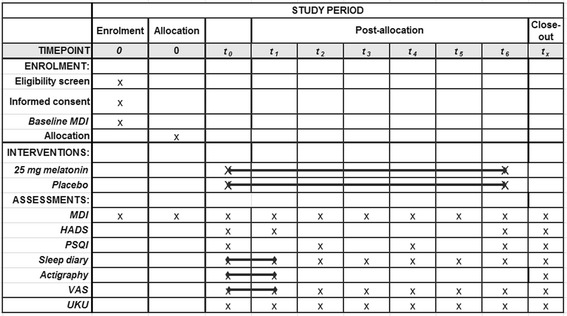
MElatonin on Depressive symptoms, Anxiety, CIrcadian and Sleep disturbances (MEDACIS) Standard Protocol Items: Recommendations for Interventional Trials (SPIRIT) figure. SPIRIT figure for the MEDACIS trial

The MEDACIS trial is a multicenter, double-blinded, placebo-controlled, randomized clinical trial investigating the effect of 25 mg exogenous melatonin (intervention group) against placebo (control group) and the study is designed as a parallel-group trial. The participants will be followed for a period of 12 weeks during the trial and assessed on outcomes continuously throughout the period (Figs. 2 and 3).

### Study setting

The study is performed in Region Zealand of Denmark, and patients will be recruited from three cardiology departments (Department of Cardiology at Holbaek Hospital, Roskilde Hospital and Koege Hospital in Region Zealand, Denmark) within the region. Roskilde Department of Cardiology has the function of performing coronary angiography (CAG) and, if necessary, percutaneous coronary intervention (PCI) in the region. A subset of the patients will have their CAG and PCI performed at the Department of Cardiology, Rigshospitalet, Copenhagen, Denmark, but will be admitted to their regional hospital afterwards. A list of recruiting sites can be found at www.clinicaltrial.gov with Identifier NCT02451293.

### Eligibility criteria

Inclusion criteria

1. Patients should be admitted to a coronary care unit for ACS [46], and should be enrolled at the latest 4 weeks after the primary ACS
2. Participants should be aged 18 years or older
3. No sign of current depression on the Major Depression Inventory (MDI) used as a diagnostic tool at the point of enrollment
4. Participants must sign an Informed Consent Form.
5. Premenopausal women (defined as no menstruation during the last 12 months) should have a negative pregnancy test. Furthermore, women of reproductive age should use reliable birth control (intrauterine devices, hormonal contraceptives including oral pills, patches, vaginal rings, and injections) during the entire trial period

Exclusion criteria

1. Known allergic reaction to melatonin
2. Ongoing or previous pharmacologically treated depression or bipolar disorder
3. No dementia as determined by a Mini Mental State Examination (MMSE) score <24 is allowed
4. At the point of inclusion no participation in other pharmacological intervention trials is allowed
5. No participants with a diagnosis of Rotor syndrome or Dubin-Johnson syndrome, epilepsy, sleep apnea syndrome, systemic lupus erythematosus (SLE), rheumatoid arthritis (RA) or multiple sclerosis are allowed
6. Severe liver disease, defined as transaminases above three times normal levels, and severe kidney disease defined as an eGRF under 40 ml/min
7. Chronic hypnotic treatment previous to the ACS
8. Known sleep disorder (e.g., insomnia, restless legs, etc.)
9. Work involving nightshifts
10. Daily alcohol consumption above 5 units of alcohol (1 unit = 12 g alcohol)
11. Predictable poor compliance (not speaking fluent Danish)
12. Pregnant or breastfeeding women
13. Severe, life-threatening medical condition that implies that the patient cannot participate in the study course (e.g., cancer, stroke)
14. Indication for coronary artery bypass graft (CABG)

### Interventions

Patients will receive either 25 mg exogenous melatonin (intervention group) or matching placebo (control group) in an orally administered pill. Regarding the active substance each pill will contain 12.5 mg melatonin, and the placebo pill will be comparable to study medication except for the active substance. The medication will be packaged in blister packs consisting of 30 pills in each, and a total of seven blister packages will be packaged in a cardboard box (total of 210 pills). Patients will be instructed to take two pills (daily dose of 25 mg active substance) approximately 1 h before bedtime during the entire length of the study (12 weeks). Medicine compliance will be investigated at weeks 2 and 12 at clinical visits. All study medication will be produced by PharmaNord Aps. (Vejle, Denmark) in accordance with Good Manufacturing Procedure (GMP). The study medication will be shipped to the Region Zealand Hospital Pharmacy which will handle the randomization process.

If a participant withdraws their consent, they will leave the study. In the unlikely event that a female participant should become pregnant despite using reliable birth control, she will leave the study. Patients who have a recurrence of ACS during the study will be withdrawn from the study. In case of severe medical emergency (e.g., stroke or severe sepsis) requiring emergency surgery, stay in an intensive care unit, or more than 1 week of hospitalization will result in exclusion from the study. Diagnosis of a cancer during the study period will also result in withdrawal from the study. No further data will be collected from patients after leaving. However, previously collected data will be part of the final data analysis. No change in dose of study medication will be possible during the trial.

A medicine compliance of a minimum of 75% (pill count) at the clinical visits at weeks 2 and 12 are required. If the patients do not comply with these demands at either visit, they will be excluded from the study. Exclusion from the study entails stopping the study medication, and patient’s data will not be part of a per-protocol analysis. In case of medicine compliance at the first clinical visit, the data collected until this point will be part of a per-protocol analysis.

If a patient is diagnosed with a moderate-to-severe depression on the MDI during the study, they will be referred to a psychiatrist in training attached to the study (who is blinded to the allocated treatment). The psychiatrist in training will perform a structured clinical interview (Hamilton Depression Scale) with the purpose of diagnosing depression and its severity according to the *International Classification of Diseases, version 10* (ICD-10) criteria. Patients with a moderate-or-severe depressive episode according to the Hamilton Depression Scale and clinical assessment will be withdrawn from the study and offered open treatment for depression via the patient’s general practitioner (GP). All outcome data collected until the eventual termination in the study will be part of the final analysis. If the patient is diagnosed with a mild depression on the MDI, they will be allowed to continue in the study. The patient will be monitored using the MDI and phone calls according to the study protocol. In case of symptom progression on the MDI into moderate to severe depression, the participant will be referred to the psychiatrist in training as described above.

Patients will be contacted by phone seven times (every 2 weeks) during the trial to assure compliance and to promote their retention during follow-up. The patients will be reminded to fill in the relevant study questionnaires, reminded to take study medication, and monitored for occurrence of any adverse reactions, or changes in other medication since last contact with study investigators.

The participants are allowed to participate in all offered concomitant care at their regional cardiology department (e.g., cardiac rehabilitation). Startup of any medical antidepressant treatment in the trial period is prohibited and will lead to exclusion from the trial.

### Outcomes

**Table:** 

Primary outcome	Exploratory outcomes
Major Depression Inventory (MDI)	Pattern of dropouts and withdrawals from the study Safety, side effect and compliance to study medication Anxiety and depression measured by the Hospital Anxiety and Depression Scale (HADS) Sleep and circadian outcomes measured by actigraphy Subjective sleep quality measured by the Pittsburgh Sleep Quality Index (PSQI) Sleep diary Sleep, pain, anxiety, fatigue and general wellbeing measured by visual analog scales (VAS) Blood samples – Circadian clock gene analysis

### Primary outcome

The MDI is a self-rating scale with 12 questions. It has been extensively used in a Danish population [47] and is also thoroughly validated [48]. The questions cover the ten ICD-10 symptoms for depression, which are identical to the *Diagnostic and Statistical Manual of Mental Disorders, 4th edition* (DSM-IV) major depression diagnostic criteria except for one symptom, low self-esteem (question 4), which in DSM-IV is incorporated into a question about guilt (question 5).

The MDI holds a double function. It can be used as a diagnostic tool which, via an algorithm, can be transformed into the DSM-IV or ICD-10 categories of “mild,” “moderate,” or “severe” depression. The tool has been found to have satisfactory sensitivity and specificity with regard to the diagnosis of depression according to both the ICD-10 and DSM-IV [49]. The MDI can also be used as a rating scale, which can grade the severity of depression and this has been found to have an acceptable correlation with the Hamilton Depression Scale (HAM-D) [48].

For the purpose of the current study, the MDI will be used as a diagnostic tool at baseline with regard to the eligibility (inclusion/exclusion criteria) of the participants. Mild depression excludes participation and, as a diagnostic tool, mild depression is the presence of two core symptoms and two accompanying symptoms of depression. During the study, the MDI will be used as a rating scale to evaluate the severity and development of depressive symptoms. As a rating scale, a total MDI score (0–50 points) of 21 to 25 points corresponds to a mild depression, of 26 to 30 a moderate depression and above 31 points constitutes a severe depression. Incident depressive symptoms, defined as MDI score ≥21 any time after baseline MDI, is the primary outcome assessed using the MDI as a rating scale. Timing of outcome assessment can be seen in the SPIRIT figure (Fig. 3).

### Exploratory outcomes

The pattern of participants leaving the study before completion of the 12-week period will be investigated. The different reasons for leaving the study (e.g., withdrawal of consent, medicine compliance, recurrent ACS, etc.) will be compared between the two groups. Furthermore, the timing of dropout between the two groups will be compared.

Safety and potential side effects will be closely monitored during the entire trial and reported in the proceeding publication. For specifics, see the “Harms and adverse events” section.

The Hospital Anxiety and Depression Scale (HADS) was originally developed by Zigmond and Snaith in 1978 [50] with the purpose of developing an easy-to-use tool for screening for mood disorders in a hospital setting. In a cardiac setting HADS-D has been shown to have adequate internal consistency, reliability and construct validity [51].

Actigraphy is a noninvasive method used to objectively evaluate a patient’s sleep and circadian rhythm. An actigraph (Octagonal Basic Motionlogger, Ambulatory Monitoring Inc., Ardsley, NY, USA), which is a wrist-worn minicomputer, will be mounted on the participant’s nondominant wrist at enrollment and worn for 2–3 weeks until the first clinical visit. Actigraphy has been shown to have a high validity and accuracy compared to polysomnography (PSG), the “gold standard” of sleep evaluation [52]. Actigraphy has been used extensively in patients undergoing surgery [53] and in cancer patients [54] but has not been investigated thoroughly in post-ACS patients [20].

Subjective sleep outcomes will be evaluated using a sleep diary, visual analog scales (VAS), and the Pittsburgh Sleep Quality Index (PSQI). A sleep diary is the patient’s own account of sleep data, and the patient is asked to fill in a diary page each morning after awakening. Visual analog scales regarding sleep will also be assessed on the scale ranging from “best conceivable sleep” at 0 mm to “worst conceivable sleep” at 100 mm. Furthermore, patients will fill out VAS on general wellbeing, fatigue, pain and anxiety. Lastly, the patients will fill out the PSQI, which assesses sleep quality during the prior 4 weeks and has a clinically established cutoff of ≥5 as being a poor sleeper and ≥8 as having sleep problems needing treatment [55].

Blood analysis will analyze for circadian genotype *PER 3* which has a variable number tandem repeat (VNTR) polymorphisms (unique to primates) that consist of either a 4- or 5-U repeated coding region. *PER 3* has been associated with sleep disorders [56], affective disorders [57] and reduced cognitive function in relation to sleep deprivation [58]. We will investigate a possible relationship between circadian genotype (clock genes), sleep-wake cycle, and depressive symptoms following ACS.

### Participant timeline

A diagnosis of ACS is made by the attending physician according to relevant guidelines. Eligible participants (according to the inclusion and exclusion criteria) will be informed about the study by the clinical investigating team, and if possible, a later inclusion meeting will be planned. Hereby, the potential participants will receive adequate time to consider participation and the possibility of bringing an assessor. The participant will be presented to the study during their initial admission for ACS or at a later joint meeting during cardiac rehabilitation. A participant should be included within 4 weeks of their ACS. The participants’ flow through the study can be seen in the CONSORT flow diagram (Fig. 2) and the timing of outcome measurement in the SPIRIT figure (Fig. 3).

### Sample size

Sample size is calculated on the basis of a conservative assumption that 31% of patients following ACS will develop depressive symptoms [4] which we assume can be reduced to 15.5% by melatonin treatment. Power calculation is based on two-sided test and, with a power of 0.80 and a significance level of 5% (*α* = 0.05), the required sample size in each group is 116. There are no interim efficacy analyses planned. The patients will be randomized in blocks of 6 and, therefore, the study will proceed until 120 patients have been enrolled in each arm.

### Recruitment

To assure adequate recruitment to the trial, the study will take place at three departments simultaneously. Recruitment from three departments should provide sufficient participants to ensure the target sample size. In case of insufficient recruitment further recruiting centers will be sought and included if possible.

### Assignment of interventions

The randomization process will be handled by the regional pharmacy in Region Zealand, Denmark. The randomization list/sequence will be made before the study start by using a dedicated online software (http://www.randomization.com/) by the regional pharmacy. Block randomization, in blocks of 6, will be used. In each block, half the patients will receive the intervention drug (melatonin 25 mg), and the other half the placebo. The randomization list will be kept at the regional pharmacy and will not be available to the investigators recruiting the participants.

Allocation concealment consists of two sets of coded envelopes (an opaque, sealed envelope for each patient, containing the randomization code for each patient) which will be produced by the Regional Pharmacy of Region Zealand. The two envelopes will be sent to Koege Hospital (sponsor’s center) and stored there; one in the patients Case Report Form (CRF), and the other in the Trial Master File (TMF). In this way, the blinding will be conserved for both the sponsor/investigator and other investigators who enroll and assign treatments to the participants.

After assignment into the trial, the trial participant, investigators, care providers, outcome assessors and data analysts will be blinded to the allocated treatment. In case of a suspected unexpected serious adverse reaction (SUSAR), unblinding can be performed by the investigators using the coded envelopes available in the patient’s CRF.

### Data collection

Data are collected in participant-specific Case Report Forms (CRFs) and will be stored for a 5-year period after study termination. As standard, an electronic CRF (eCRF) will be used to collect all study data from each participant throughout the study. The eCRF is created using the online platform Easytrial.net (Easytrial ApS, Glostrup, Denmark) which serves as the electronic data storage of the clinical trial. The individual questionnaires are sent to the individual participants via email with a unique generated link. In case a participant is noncompliant with the electronic setup (i.e., no Internet connection), they will fill out study questionnaires in paper format and the data will be transferred manually to the online study database. At the point of inclusion, baseline demographics will be collected from the patient and the patient record.

As previously described, phone calls and electronic (e.g., email) reminders will be sent out to assure participant retention and reduce loss to follow-up. No additional data are to be collected if a participant discontinues study medication or in other ways does not comply with the intervention protocols.

Using the eCRF implies that answered questionnaires are source data and are transferred directly to the electronic database upon completion. The sleep diary and the VAS administered for the initial 2 weeks are in paper format, and will later be transferred to the electronic database using double data entry. The study will be performed in accordance with Good Clinical Practice (GCP) and monitored by the GCP Unit from the University of Copenhagen. After completion of the trial the sponsor/investigator (first author) will have access to the final trial dataset.

### Statistics

Descriptive data on baseline characteristics and results will be presented as mean, SD, and *n* and presented in tabular form. Distribution of data will be tested by using data plots and by visually assessing data, and potentially continuous variables will be visualized in a matrix plot. Appropriate statistical tests will be used accordingly depending on distribution. Log transformation will be applied if applicable.

Our null hypothesis is that there will be no difference in the number of participants developing depressive symptoms (defined as a MDI score ≥21) throughout the study period between the melatonin and the placebo group. The hypothesis is assessed using Fisher’s exact test and/or the chi-squared test.

For the primary outcome analysis, a linear mixed model will be applied. The effect of age, gender, comorbidity and cardiac risk factors will be assessed by marginal analysis. Results will be assessed through an analysis on complete data generated using multiple imputation.

As a secondary analysis, the cumulative incidence of depressive symptoms will be plotted for the two groups and compared using the log-rank test assuming the absence of competing risks. Incidence of depressive symptoms (MDI score ≥21) will be analyzed using the Cox proportional hazards regression, including analysis of effects of age, gender, comorbidity and cardiac risk factors at baseline.

A principle analysis will be performed according to the intention-to-treat principle (ITT). The intention-to-treat population is defined as every patient randomized into the trial. A per-protocol analysis will also be performed. A per-protocol participant is defined as a patient who has taken medication in accordance with the protocol (at least 75%) for the first 14 days (until the follow-up visit). In cases of noncompliance with the medication before the follow-up visit (i.e., exclusion from the study), another patient will be enrolled to make up for the lost patient. Finally, a standard sensitivity analysis will be performed on the per-protocol patient population.

For secondary/exploratory outcomes, intergroup comparison for anxiety and depression (HADS) will be made at baseline, 2 and 12 weeks. The data originating from the actigraphy measurement will be presented as sleep and circadian outcomes. The sleep outcomes will be divided into units of 3 days as recommended [59], and descriptive data will be presented for the entire 2-week measuring period. Sleep diary and VAS measured each day simultaneously with actigraphy will be termed short-term sleep/VAS outcomes. Sleep diary and VAS measured afterwards every 2 weeks will be termed long-term sleep/VAS outcomes. Intergroup comparisons of PSQI data will be made at baseline, 4, 8 and 12 weeks. For all continuous data, intergroup comparisons will be done using an unpaired Student *t* test or Mann-Whitney’s test depending on distribution of data. For analysis of the correlation between circadian genotype, depressive symptoms and sleep, a logistic regression analysis will be used.

A *P* value < 0.05 will be considered statistically significant. For exploratory outcomes all analysis will be presented and no statistical correction for multiple testing will be applied [60]. No interim analysis is planned for the study. A priori, no subgroup analysis is planned [61]. The statistical analysis plan has been consulted with an external independent statistician. The analysis of data will, upon study completion, be handled and performed by the first author, and the process will be aided by the external study statistician. An appropriate statistical software package will be applied for the statistical analysis.

### Harms and adverse events

To monitor harms and adverse events two systems will be employed in the current trial. Firstly, at each clinical visit and during each phone call the patient will be interviewed regarding possible side effects according to the summary of product characteristics which contains a list of known side effects. Furthermore, an open-ended question regarding side effects will be asked to try to detect any unexpected side effects.

Secondly, the “udvalg for klinisk undersøgelser” (UKU) manual will be used to monitor harms. The UKU has been developed for monitoring the side effects of psychotropic drugs and has been validated in several Nordic countries [62]. A self-rating version of the UKU side effect rating scale (UKU-SERS-Pat) has been developed and validated against the clinician-administered version (UKU-SERS-Clin) [63]. The UKU-SERS-Clin is time-consuming which may hinder its usage in clinical practice with the possibility of overlooking severe side effects that may be avoided. Furthermore, development of a patient-administered version may gain more information about side effects as patients and clinicians perceive them differently [63]. Overall, the intercorrelation between the patient- and clinician-administered UKU was shown to be statistically significant [63]. When working with antidepressants, the UKU-SERS paper [62] states that neurological symptoms may be omitted from the evaluation, and this will be done in the current study. In the current clinical trial, the UKU-SERS-Pat will be administered at the clinical visits and in the ambulatory setting at the point in the trial when the clinical status (MDI) is being evaluated.

### Ethics, monitoring and reporting

The study will be performed in agreement with the Helsinki II Declaration and law 593 of 2011 pertaining to the Scientific Ethics Committee System. The project is registered at www.ClinicalTrials.gov (NCT02451293) as recommended by the International Committee of Medical Journal Editors (ICMJE). The Good Clinical Practice Unit at Copenhagen University will oversee the trial and conduct monitoring visits of the trial periodically. The protocol has followed the SPIRIT guidelines (Additional file 1), and the manuscript will follow the CONSORT Statement [64]. In accordance with CONSORT guidelines, both positive, neutral and negative findings will be published [64]. In case of important modifications to the protocol (e.g., changes in eligibility criteria), trial investigators will be informed and the www.ClinicalTrials.gov registration updated. Authorship guidelines put forth by the ICMJE will be applied for all publications originating from the current trial.

## Discussion

The current manuscript describes a protocol for a primary prophylactic study targeting nondepressed patients following ACS. To the best of our knowledge, we are the first to investigate melatonin’s antidepressant, anxiolytic and hypnotic effects in this population.

The predominant focus of previous trials has been the treatment of depression in patients following ACS. A recent Danish study showed that 20% of patients following ACS have an incident depression and 67% of medical antidepressant treatment is made up of selective serotonin reuptake inhibitor (SSRIs) [65]. This is coherent with a systematic review describing the increased use of antidepressants (predominantly SSRIs) during the last three decades [66]. Awareness and treatment of depression following ACS has risen during the last decades; however, not much attention has been focused on preventive interventions.

We know of only one previous trial using a similar primary preventive strategy [67, 68], which showed an effect of prophylactic treatment with escitalopram (Cipralex) [67]. However, treating patients with a SSRI (SSRI before the onset of depression is not without risk. Treatment with SSRIs have been shown to be associated with sleep disturbances, sexual disturbances and arrhythmias [30]. Recently, specific concerns regarding the cardiotoxicity of citalopram (also escitalopram) have been raised [69]. There exists a dose-response relationship between citalopram and the prolonged QT interval syndrome which, in some cases, leads to sudden cardiac death. Furthermore, patients with a previous cardiac event (e.g., MI) constitute a high-risk population [69]. The DECARD trial [67] has, in light of this, been criticized for the unnecessary potential harm associated with SSRI treatment caused to nondepressed patients with a previous cardiac event. The premise of preventing the development of depressive symptoms in the specific population is, however, controversial. Any such intervention should, of course, have the highest degree of benefit and the lowest incidence of side effects.

Melatonin was chosen for the current trial in light of these considerations, and on the basis of positive results from a previous similar trial in women with breast cancer [37]. It should be mentioned that the study did not include the planned number of patients due to slow recruitment; however, the effect of melatonin was greater than assumed a priori [37]. Melatonin has repeatedly been shown to have sleep-improving effects in women with breast cancer [38, 70, 71]. As melatonin is widely safe and nontoxic, even when given to animals and humans in high exogenous doses over extended time periods [35, 45, 72], it would be a good candidate for a prophylactic setup. Exogenous melatonin has been investigated in multiple clinical trials and with multiple outcomes [31, 43]. Melatonin has recently been reviewed as treatment for clinical depression in both prophylactic and treatment setups [43], where no significant antidepressant effect of melatonin was shown in either setting. However, it was concluded that the included studies were heterogonous and of low quality, and that further properly dimensioned trials will be needed before any conclusion can be made. The review does not include the previously mentioned positive prophylactic melatonin trial performed in breast cancer patients [37]. Furthermore, the included trials have used a low dose of melatonin, typically under 10 mg [43] which is the dose used for treatment of insomnia in patients aged over 55 years [44]. It has been argued by Cardinali et al. that the antidepressant potential of melatonin cannot be determined before trials using equivalent doses of melatonin compared to other melatonergic agonists [30] have been performed. This would constitute trials of 25–100 mg melatonin, and taking into consideration the prophylactic setup of our trial, we chose a dose of 25 mg. In a dose-response study, a dose of 25 mg Valdoxan (agomelatine, a melatonin agonist) was found to be most effective as an antidepressant [73], and 25 mg is typically the recommended induction dose for Valdoxan in treatment of major depressive disorders. To the best of our knowledge, a similar dose-response trial does not exist for melatonin in relation to its antidepressant effect. Twenty-five milligrams is, therefore, arbitrarily chosen, but it is never-the-less the dose chosen in light of the dose-response antidepressant effects seen in melatonin agonists.

Besides the positive effect on depressive symptoms, melatonin may also be used to treat anxiety, sleep and circadian disturbances. Melatonin has been recommended as the first-line treatment of patients aged over 55 years suffering from insomnia, parasomnia and circadian rhythm sleep disorders [44]. Exogenous melatonin administered in the perioperative period has recently been reviewed [31], showing a significant anxiolytic and analgesic effect. Overall, melatonin is a relevant and safe intervention to be tested for potentially beneficial effects against depressive symptoms, anxiety and sleep and circadian disturbances in patients following ACS.

### Status of the trial

The study gained all relevant approvals during the end of 2015, and permission to initiate the trial was gained on 20 January 2016. The trial was initiated firstly at the Departments of Cardiology at Zealand University Hospital (Koege), followed by Zealand University Hospital (Roskilde) and lastly Holbaek Hospital.

The trial has at this point included 150 out of 240 patients.

## Additional file

Additional file 1: Checklist: recommended items to address in a clinical trial protocol and related documents. (DOC 123 kb)

## Abbreviations

ACS: Acute coronary syndrome
AHA: American Heart Association
BDI: Beck Depression Inventory
CABG: Coronary artery bypass graft
CAG: Coronary angiography
CBT: Cognitive behavioural therapy
CRF: Case Report Form
DSM-IV: *Diagnostic and Statistical Manual of Mental Disorders, 4th edition*
eGFR: Estimated glomerular filtration rate
GMP: Good Manufacturing Practice
HADS: Hospital Anxiety and Depression Scale
HAM-D: Hamilton Depression Scale
ITT: Intention-to-treat principle
MDI: Major Depression Inventory
MEDACIS: The effect of MElatonin and Depression, Anxiety, CIrcadian and Sleep disturbances in patients after acute coronary syndrome
MI: Myocardial infarction
MMSE: Mini Mental State Examination score
PCI: Percutaneous coronary intervention
PSQI: Pittsburgh Sleep Quality Index
RA: Rheumatoid arthritis
SLE: Systemic lupus erythematosus
SSRI: Selective serotonin reuptake inhibitor
TMF: Trial Master File
UKU-SERS-Clin: UKU side effect rating scale clinician-administered version
UKU-SERS-Pat: UKU side effect rating scale patient version
VAS: Visual analog scales

## References

[1] Mathers CD, Loncar D (2006). Projections of global mortality and burden of disease from 2002 to 2030. PLoS Med.

[2] White HD, Chew DP (2008). Acute myocardial infarction. Lancet.

[3] Koch MB, Johnsen NF, Davidsen M, Juel K. Hjertekarsygdomme i 2011 incidens, prævalens og dødelighed samt udvikling siden 2002. 5-1-2014. http://www.si-folkesundhed.dk/upload/hjertekarsygdomme_i_2011-2_rapport.pdf. Accessed 3 Feb 2017. Statens Institut for Folkesundhed, Syddansk Universitet for Hjerteforeningen. Ref Type: Report.

[4] Thombs BD, Bass EB, Ford DE, Stewart KJ, Tsilidis KK, Patel U (2006). Prevalence of depression in survivors of acute myocardial infarction. J Gen Intern Med.

[5] Jiang W, Krishnan RR, O’Connor CM (2002). Depression and heart disease: evidence of a link, and its therapeutic implications. CNS Drugs.

[6] Lichtman JH, Froelicher ES, Blumenthal JA, Carney RM, Doering LV, Frasure-Smith N (2014). Depression as a risk factor for poor prognosis among patients with acute coronary syndrome: systematic review and recommendations: a scientific statement from the American Heart Association. Circulation.

[7] Meijer A, Conradi HJ, Bos EH, Thombs BD, van Melle JP, de Jonge P (2011). Prognostic association of depression following myocardial infarction with mortality and cardiovascular events: a meta-analysis of 25 years of research. Gen Hosp Psychiatry.

[8] Lichtman JH, Bigger JT, Blumenthal JA, Frasure-Smith N, Kaufmann PG, Lesperance F (2008). Depression and coronary heart disease: recommendations for screening, referral, and treatment: a science advisory from the American Heart Association Prevention Committee of the Council on Cardiovascular Nursing, Council on Clinical Cardiology, Council on Epidemiology and Prevention, and Interdisciplinary Council on Quality of Care and Outcomes Research: endorsed by the American Psychiatric Association. Circulation.

[9] Mavrides N, Nemeroff C (2013). Treatment of depression in cardiovascular disease. Depress Anxiety.

[10] Strik JJ, Honig A, Lousberg R, Denollet J (2001). Sensitivity and specificity of observer and self-report questionnaires in major and minor depression following myocardial infarction. Psychosomatics.

[11] Denollet J, Strik JJ, Lousberg R, Honig A (2006). Recognizing increased risk of depressive comorbidity after myocardial infarction: looking for 4 symptoms of anxiety-depression. Psychother Psychosom.

[12] Lane D, Carroll D, Ring C, Beevers DG, Lip GY (2002). The prevalence and persistence of depression and anxiety following myocardial infarction. Br J Health Psychol.

[13] Mayou RA, Gill D, Thompson DR, Day A, Hicks N, Volmink J (2000). Depression and anxiety as predictors of outcome after myocardial infarction. Psychosom Med.

[14] Roest AM, Martens EJ, Denollet J, de Jonge P (2010). Prognostic association of anxiety post myocardial infarction with mortality and new cardiac events: a meta-analysis. Psychosom Med.

[15] Celano CM, Millstein RA, Bedoya CA, Healy BC, Roest AM, Huffman JC (2015). Association between anxiety and mortality in patients with coronary artery disease: a meta-analysis. Am Heart J.

[16] Watkins LL, Blumenthal JA, Carney RM (2002). Association of anxiety with reduced baroreflex cardiac control in patients after acute myocardial infarction. Am Heart J.

[17] Young EA, Abelson JL, Cameron OG (2004). Effect of comorbid anxiety disorders on the hypothalamic-pituitary-adrenal axis response to a social stressor in major depression. Biol Psychiatry.

[18] von Känel R, Kudielka BM, Schulze R, Gander ML, Fischer JE (2004). Hypercoagulability in working men and women with high levels of panic-like anxiety. Psychother Psychosom.

[19] Grace SL, Abbey SE, Irvine J, Shnek ZM, Stewart DE (2004). Prospective examination of anxiety persistence and its relationship to cardiac symptoms and recurrent cardiac events. Psychother Psychosom.

[20] Coryell VT, Ziegelstein RC, Hirt K, Quain A, Marine JE, Smith MT (2013). Clinical correlates of insomnia in patients with acute coronary syndrome. Int Heart J.

[21] Morin CM, Ware JC (1996). Sleep and psychopathology. Appl Prev Psychol.

[22] Ohayon MM, Roth T (2003). Place of chronic insomnia in the course of depressive and anxiety disorders. J Psychiatr Res.

[23] Lopez-Jimenez F, Sert Kuniyoshi FH, Gami A, Somers VK (2008). Obstructive sleep apnea: implications for cardiac and vascular disease. Chest.

[24] Leineweber C, Kecklund G, Janszky I, Akerstedt T, Orth-Gomer K (2003). Poor sleep increases the prospective risk for recurrent events in middle-aged women with coronary disease. The Stockholm Female Coronary Risk Study. J Psychosom Res.

[25] Srinivasan V, Pandi-Perumal SR, Trakht I, Spence DW, Hardeland R, Poeggeler B (2009). Pathophysiology of depression: role of sleep and the melatonergic system. Psychiatry Res.

[26] Carney CE, Segal ZV, Edinger JD, Krystal AD (2007). A comparison of rates of residual insomnia symptoms following pharmacotherapy or cognitive-behavioral therapy for major depressive disorder. J Clin Psychiatry.

[27] van Straten A, Geraedts A, Verdonck-de Leeuw I, Andersson G, Cuijpers P (2010). Psychological treatment of depressive symptoms in patients with medical disorders: a meta-analysis. J Psychosom Res.

[28] Hackett ML, Anderson CS, House A, Halteh C. Interventions for preventing depression after stroke. Cochrane Database Syst Rev. 2008;3:CD003689. 10.1002/14651858.CD003689.pub3 18646094

[29] Claustrat B, Brun J, Chazot G (2005). The basic physiology and pathophysiology of melatonin. Sleep Med Rev.

[30] Cardinali DP, Srinivasan V, Brzezinski A, Brown GM (2012). Melatonin and its analogs in insomnia and depression. J Pineal Res.

[31] Andersen LP, Werner MU, Rosenberg J, Gogenur I. A systematic review of peri-operative melatonin. Anaesthesia. 2014;69(10):1163–71. 10.1111/anae.12717 24835540

[32] Arendt J, Skene DJ (2005). Melatonin as a chronobiotic. Sleep Med Rev.

[33] Brzezinski A, Vangel MG, Wurtman RJ, Norrie G, Zhdanova I, Ben-Shushan A (2005). Effects of exogenous melatonin on sleep: a meta-analysis. Sleep Med Rev.

[34] Yang Y, Sun Y, Yi W, Li Y, Fan C, Xin Z (2014). A review of melatonin as a suitable antioxidant against myocardial ischemia-reperfusion injury and clinical heart diseases. J Pineal Res.

[35] Zhdanova IV (2005). Melatonin as a hypnotic: pro. Sleep Med Rev.

[36] Reiter RJ, Tan DX, Osuna C, Gitto E (2000). Actions of melatonin in the reduction of oxidative stress. A review. J Biomed Sci.

[37] Hansen MV, Andersen LT, Madsen MT, Hageman I, Rasmussen LS, Bokmand S (2014). Effect of melatonin on depressive symptoms and anxiety in patients undergoing breast cancer surgery: a randomized, double-blind, placebo-controlled trial. Breast Cancer Res Treat.

[38] Madsen MT, Hansen MV, Andersen LT, Hageman I, Rasmussen LS, Bokmand S, et al. Effect of melatonin on sleep in the perioperative period after breast cancer surgery: a randomized, double-blind, placebo-controlled trial. J Clin Sleep Med. 2016;12(2):225–33. 10.5664/jcsm.5490 PMC4751412 26414973

[39] Hansen MV, Madsen MT, Andersen LT, Hageman I, Rasmussen LS, Bokmand S (2014). Effect of melatonin on cognitive function and sleep in relation to breast cancer surgery: a randomized, double-blind, placebo-controlled trial. Int J Breast Cancer.

[40] Ferracioli-Oda E, Qawasmi A, Bloch MH (2013). Meta-analysis: melatonin for the treatment of primary sleep disorders. PLoS One.

[41] Buscemi N, Vandermeer B, Hooton N, Pandya R, Tjosvold L, Hartling L (2006). Efficacy and safety of exogenous melatonin for secondary sleep disorders and sleep disorders accompanying sleep restriction: meta-analysis. BMJ.

[42] Hansen MV, Halladin NL, Rosenberg J, Gogenur I, Moller AM (2015). Melatonin for pre- and postoperative anxiety in adults. Cochrane Database Syst Rev.

[43] Hansen MV, Danielsen AK, Hageman I, Rosenberg J, Gogenur I (2014). The therapeutic or prophylactic effect of exogenous melatonin against depression and depressive symptoms: a systematic review and meta-analysis. Eur Neuropsychopharmacol.

[44] Wilson SJ, Nutt DJ, Alford C, Argyropoulos SV, Baldwin DS, Bateson AN (2010). British Association for Psychopharmacology consensus statement on evidence-based treatment of insomnia, parasomnias and circadian rhythm disorders. J Psychopharmacol.

[45] Andersen LP, Gogenur I, Rosenberg J, Reiter RJ (2016). The safety of melatonin in humans. Clin Drug Investig.

[46] Thygesen K, Alpert JS, Jaffe AS, Simoons ML, Chaitman BR, White HD (2012). Third universal definition of myocardial infarction. J Am Coll Cardiol.

[47] Olsen LR, Mortensen EL, Bech P (2004). Prevalence of major depression and stress indicators in the Danish general population. Acta Psychiatr Scand.

[48] Olsen LR, Jensen DV, Noerholm V, Martiny K, Bech P (2003). The internal and external validity of the Major Depression Inventory in measuring severity of depressive states. Psychol Med.

[49] Bech P, Rasmussen NA, Olsen LR, Noerholm V, Abildgaard W (2001). The sensitivity and specificity of the Major Depression Inventory, using the Present State Examination as the index of diagnostic validity. J Affect Disord.

[50] Zigmond AS, Snaith RP (1983). The Hospital Anxiety and Depression Scale. Acta Psychiatr Scand.

[51] Thombs BD, Magyar-Russell G, Bass EB, Stewart KJ, Tsilidis KK, Bush DE (2007). Performance characteristics of depression screening instruments in survivors of acute myocardial infarction: review of the evidence. Psychosomatics.

[52] Sadeh A (2011). The role and validity of actigraphy in sleep medicine: an update. Sleep Med Rev.

[53] Madsen MT, Rosenberg J, Gogenur I (2013). Actigraphy for measurement of sleep and sleep-wake rhythms in relation to surgery. J Clin Sleep Med.

[54] Madsen MT, Huang C, Gogenur I. Actigraphy for measurements of sleep in relation to oncological treatment of patients with cancer: a systematic review. Sleep Med Rev. 2015;20:73–83. 10.1016/j.smrv.2014.07.002 25155183

[55] Carpenter JS, Andrykowski MA (1998). Psychometric evaluation of the Pittsburgh Sleep Quality Index. J Psychosom Res.

[56] Archer SN, Robilliard DL, Skene DJ, Smits M, Williams A, Arendt J (2003). A length polymorphism in the circadian clock gene Per3 is linked to delayed sleep phase syndrome and extreme diurnal preference. Sleep.

[57] Artioli P, Lorenzi C, Pirovano A, Serretti A, Benedetti F, Catalano M (2007). How do genes exert their role? Period 3 gene variants and possible influences on mood disorder phenotypes. Eur Neuropsychopharmacol.

[58] Dijk DJ, Archer SN (2010). PERIOD3, circadian phenotypes, and sleep homeostasis. Sleep Med Rev.

[59] Littner M, Kushida CA, Anderson WM, Bailey D, Berry RB, Davila DG (2003). Practice parameters for the role of actigraphy in the study of sleep and circadian rhythms: an update for 2002. Sleep.

[60] Schulz KF, Grimes DA (2005). Multiplicity in randomised trials I: endpoints and treatments. Lancet.

[61] Schulz KF, Grimes DA (2005). Multiplicity in randomised trials II: subgroup and interim analyses. Lancet.

[62] Lingjaerde O, Ahlfors UG, Bech P, Dencker SJ, Elgen K (1987). The UKU side effect rating scale. A new comprehensive rating scale for psychotropic drugs and a cross-sectional study of side effects in neuroleptic-treated patients. Acta Psychiatr Scand Suppl.

[63] Lindstrom E, Lewander T, Malm U, Malt UF, Lublin H, Ahlfors UG (2001). Patient-rated versus clinician-rated side effects of drug treatment in schizophrenia. Clinical validation of a self-rating version of the UKU Side Effect Rating Scale (UKU-SERS-Pat). Nord J Psychiatry.

[64] Schulz KF, Altman DG, Moher D (2010). CONSORT 2010 Statement: updated guidelines for reporting parallel group randomised trials. Trials.

[65] Osler M, Martensson S, Wium-Andersen IK, Prescott E, Andersen PK, Jorgensen TS (2016). Depression after first hospital admission for acute coronary syndrome: a study of time of onset and impact on survival. Am J Epidemiol.

[66] Czarny MJ, Arthurs E, Coffie DF, Smith C, Steele RJ, Ziegelstein RC (2011). Prevalence of antidepressant prescription or use in patients with acute coronary syndrome: a systematic review. PLoS One.

[67] Hansen BH, Hanash JA, Rasmussen A, Hansen JF, Andersen NL, Nielsen OW (2012). Effects of escitalopram in prevention of depression in patients with acute coronary syndrome (DECARD). J Psychosom Res.

[68] Hansen BH, Hanash JA, Rasmussen A, Hansen JF, Birket-Smith M (2009). Rationale, design and methodology of a double-blind, randomized, placebo-controlled study of escitalopram in prevention of Depression in Acute Coronary Syndrome (DECARD). Trials.

[69] Cooke MJ, Waring WS (2013). Citalopram and cardiac toxicity. Eur J Clin Pharmacol.

[70] Chen WY, Giobbie-Hurder A, Gantman K, Savoie J, Scheib R, Parker LM (2014). A randomized, placebo-controlled trial of melatonin on breast cancer survivors: impact on sleep, mood, and hot flashes. Breast Cancer Res Treat.

[71] Innominato PF, Lim AS, Palesh O, Clemons M, Trudeau M, Eisen A, et al. The effect of melatonin on sleep and quality of life in patients with advanced breast cancer. Support Care Cancer. 2016;24(3):1097–105. 10.1007/s00520-015-2883-6 26260726

[72] Herxheimer A, Petrie KJ. Melatonin for the prevention and treatment of jet lag. Cochrane Database Syst Rev. 2002;2:CD001520. 10.1002/14651858.CD001520 12076414

[73] Loo H, Hale A, D’haenen H (2002). Determination of the dose of agomelatine, a melatoninergic agonist and selective 5-HT(2C) antagonist, in the treatment of major depressive disorder: a placebo-controlled dose range study. Int Clin Psychopharmacol.

